# Medical Assurance Annuity Society

**Published:** 1888-02-11

**Authors:** 


### MEDICAL ASSURANCE ANNUITY SOCIETY.

The summary of the general results of the work of the Medical Sickness, Annuity, and Life Assurance Society for 1887 has just been prepared under the Friendly Societies Act for transmission to the Chief Registrar. The results are in many respects remarkable, as showing the singular success with which this Society has achieved an object which had hitherto baffled the leading assurance societies, namely, the provision of a sickness fund making adequate payments to the professional classes for permanent or temporary disability from sickness and accident, as well as the customary provision for annuity and life assurance. From this it appears that the Society commenced this its third year of active operation, with an available membership of 728; 122 members had joined during the year; 3 had died, and 18 lapsed or withdrawn; leaving a net membership of 829, or an increase of 101 in the year. The total income to the benefit or assurance fund (including £491 16*s.* 7*d.* interest on reserves) was £8,083 13*s.* as against an expenditure of £2,010 19*s.* 8*d.*, showing an increase for the year of £6,072 14*s.*, and a total reserve to these funds at the end of the year of £19,118 1*s.* 5*d.* The annual income from management purposes had been £911 17*s.* 6*d.*, of which, however, only £404 17*s.* 4*d.* had been expended, leaving a gain placed to the credit of the general assets of £507 on the year, and a balance of saving in the management for the three years of £1,703 10*s.* 8*d.* The total expenditure for management during the year had been a little over 4 per cent. The net result, therefore, of the financial work of the year was £6,579 14*s.* 2*d.*, leaving a gross capital (the whole of which is the property of the assured) of £20,821 12*s.* 1*d.* These results are, so far as is known, unparalleled in the insurance world in respect to the remarkable economy of management, the rapid and satisfactory growth of assets as against liabilities, and the complete demonstration which they afford of the success with which the organisation provided under the Friendly Societies Act can be worked, when due economy and efficient management are applied, for the benefit of the professional classes. The medical profession owe this invaluable society mainly to the energy and perseverance of Mr. Ernest Hart, its founder, who is to be congratulated most cordially on this really surprising and encouraging success. Every general practitioner might usefully become a member of this society, the office of which is at 26, Wynne Road, Brixton, S.W.

